# Can It Be Safe and Aesthetic? An Eight-year Retrospective Review of Mastopexy with Concurrent Breast Augmentation

**DOI:** 10.1097/GOX.0000000000002272

**Published:** 2019-06-12

**Authors:** Jourdain D. Artz, Oren Tessler, Steven Clark, Shukan Patel, Radbeh Torabi, Michael Moses

**Affiliations:** From the * Department of Plastic Surgery, Louisiana State University Health Sciences Center, New Orleans, La.; † Department of Plastic Surgery, Louisiana State University Health Sciences Center, New Orleans, La.; ‡ Steven Clark Plastic and Hand Surgery, McKinney, Tex.; § Louisiana State University Health Science Center, New Orleans, La.; ¶ Department of Plastic Surgery, Louisiana State University, New Orleans, La.

## Abstract

**Background:**

The safety of concurrently performing mastopexy and breast augmentation is controversial, due to the risk of breast tissue and nipple neurovascular compromise and overall potential high complications rates. This article describes a concurrent procedure of augmentation with implants and a “Tailor-Tack” mastopexy that consistently achieves an aesthetically pleasing breast with acceptable complication rates.

**Methods:**

This is a retrospective chart review of all consecutive breast augmentations performed concurrently with mastopexy using the “Tailor-Tack” technique by the 2 senior authors (M.M. and O.T.) over an 8-year period. Independent variables were patient demographics, surgical approach, implant type, shape, size, duration of follow-up, and complications. Complications were categorized as “early” (ie, first 30 days) or “late” (ie, after 30 days). Potential early complications include hematoma, skin necrosis, infection, and nipple loss. Potential late complications include recurrent breast ptosis, poor shape of the nipple areolar complex, hypertrophic scarring, implant rupture, capsular contracture, decreased nipple sensation, implant extrusion, reoperation, and scar revisions. The key principle of the technique is to place the breast implant in the dual plane first, and then perform the tailor tacking of the skin for the mastopexy second.

**Results:**

Fifty-six consecutive patients underwent augmentation and mastopexy over 8 years with this technique. The average age of the studied patients was 41.2 years. The average follow-up time period was 2.1 years (±8.9 months). Fifty-four patients (96.4%) had implants placed through the periareolar approach, 2 patients (3.6%) had implants placed via the inframammary approach. All implants were placed in a dual plane. Fifty-two patients (92.9%) received silicone implants and 4 patients (7.1%) received saline implants. Patient preference determined implant choice. All but 5 patients had textured implants. Average implant size was 277 cm^3^ (range 120–800 cm^3^). Ten patients had complications (17.9%). Complications included hypertrophic scarring in 5 (8.9%) patients; poor nipple-areola complex shape in 4 patients (7.1%); implant ruptures in 3 patients (5.4%); capsular contracture in 3 patients (5.4%); and recurrent ptosis in 2 patients (3.6%). There were no reported early complications such as nipple loss, breast skin necrosis, decreased nipple sensation, implant infections, or extrusions. However, 6 patients (10.7%) required return trips to the operating room for revisions, and 1 patient (1.8%) had a nipple areolar complex scar revised in the office, yielding a 12.5% surgical revision rate for the late complications.

**Conclusions:**

It is safe to concurrently perform mastopexy and breast augmentation. In our 8-year review, there were no early catastrophic complications such as skin loss, nipple loss, implant extrusion, or infection. The complications that occurred were the same complications known to occur with the independent performance of mastopexy alone or breast augmentation alone, and they occurred at rates comparable to or less than the national averages for those procedures when they are performed independently. The paramount principle for the success of this technique is to first adjust breast volume and then perform an intraoperatively determined skin resection to fit the new breast volume.

## Abstract 2

Supplemental Digital Content is available in the text.

For almost 3 decades there has been substantial debate concerning the safety of concurrently performing mastopexy and breast augmentation.^1^ Skin loss, nipple malposition, decreased nipple sensation, wound dehiscence, and loss of the nipple areolar complex have been described as negative outcomes in the literature, leading some surgeons to categorically condemn the procedure.^2^ Some surgeons contend that mastopexy with augmentation has an unacceptably high complication rate; however, the joint procedure continues to grow in popularity.^2,3^

Concerns about the concurrent procedure are valid and logical. Mastopexy alone can compromise breast skin viability, nipple sensation, or nipple vascularity, because it increases tension on the breast skin envelope.^4^ Therefore, it seems intuitive that decreasing the breast skin envelope surface area while simultaneously increasing the breast volume with an implant would be problematic by producing a greater degree of nipple neurovascular stress, unpredictable scarring, and incisions closed under tension. Surgery to simultaneously alter both breast volume and skin surface area in a simple, safe, and predictable procedure is undoubtedly challenging and potentially catastrophic.

Despite these potential surgical pitfalls, many surgeons believe that mastopexy with concurrent augmentation can be performed safely and reliably with complications ranging from 13% to 36%.^5–9^ This is comparable to the reported complication rates of mastopexy alone (8.6%–33.3%)^6,9^ or augmentation mammaplasty alone (13%–36%).^6,9,10^ The obvious benefit, for both patient and surgeon, is that mastopexy with simultaneous augmentation is a single procedure, which decreases the surgical expense, amount of required anesthesia, and duration of recovery time.

Several different techniques have been described for performing mastopexy with augmentation. The classic technique of the “Inverted-T” or Wise pattern skin reduction remains the most frequently used procedure in the United States.^4^ Recently, the vertical mastopexy with augmentation has gained popularity as it leaves no transverse inframammary scar and minimizes the “bottoming out” phenomenon.^6^ The “Tailor-Tack” mastopexy is derived from the “Inverted-T” mastopexy but differs from it by custom tailoring the skin excision intraoperatively to exactly fit the new breast volume.^11^

This article will review single-stage mastopexy with breast augmentation, and it will describe the “Tailor-Tack” technique that the senior authors (O.T. and M.M.) performed on consecutive patients over an 8-year period. This technique results in a new, aesthetically pleasing breast volume and a tight skin envelope that precisely corrects excess breast skin without compromising tissue viability. It is our belief that mastopexy with augmentation using the “Tailor-Tack” method not only addresses the concerns of nipple viability and skin surface area correction but also is an acceptably safe, single-stage procedure that is equivalent in results and risks to the 2-stage techniques.

## PATIENTS AND METHODS

A retrospective chart review was performed to identify all consecutive breast augmentations that were concurrently performed with mastopexy by 2 surgeons, the senior authors (O.T. and M.M.), over an 8-year period. It was during this time that they began performing mastopexy with augmentations with the current “Tailor-Tack” technique. Independent variables examined were surgical approach, breast implant type, implant manufacturer, implant shape, implant size, any performance of additional procedures at the time of the initial surgery, duration of follow-up, and complications. Our complications were categorized as “early” (ie, first 30 days) or “late” (ie, after 30 days). Complications were subcategorized as “tissue-related” or “implant-related.” Potential tissue-related complications consisted of hematoma, skin necrosis, soft tissue infection, nipple loss, recurrent breast ptosis, poor shape of the nipple areolar complex, and hypertrophic scarring. Potential implant-related complications consisted of implant rupture, implant infections, implant extrusion, and capsular contracture.

### Preoperative Patient Evaluation

Preoperatively, each patient was asked to determine her desired breast size by identifying photographs of bare breasts that illustrated her ideal breast size. Preoperatively, trial sizers were used in the patient’s bra to estimate the volume of the implant needed to achieve her desired look. The final implant choice was determined as follows: (1) matching the width of the implant to the width of the patient’s chest; (2) determining the projection of the implant according to the patient’s desired volume; and (3) treating the height of the implant (for shaped implants) as the least important parameter.

### Augmentation Surgical Technique

On the morning of surgery, the midline of the patient’s chest, the existing inframammary folds, the midmeridian of each breast, and the approximate final nipple-areolar height were marked. Average nipple-areolar height was approximately 1–2 cm above the height of the inframammary fold in the sitting position. Nipple-areolar height was placed slightly higher for younger women and/or smaller breasted women; conversely, the height was placed slightly lower for older women and/or larger breasted women.

In surgery, the breast augmentation was performed before the mastopexy. The nipple-areolar complex was reduced to an approximately 36- to 38-mm-diameter circle centered on the nipple, de-epithelializing the remainder of the areola to its periphery. Younger women often request smaller areolae; older women and/or women with large breasts may request slightly larger areolar diameter.

Through the upper half of the de-epithelialized donut surrounding the retained areola, electrocautery was used to traverse through the breast tissue straight down to the pectoralis fascia and muscle. The muscle was divided parallel to its fibers directly beneath the areola, and a subpectoral pocket was created medially to 1½ cm from the midline, inferiorly to 1 fingerbreadth below the existing inframammary fold, and laterally to the anterior axillary line. The pectoralis major muscle was released inferiorly and medially up to the level of the nipple. Trial saline sizers were used to determine the final implant size, with the final decision being made with the patient sitting upright. Every attempt was made to match the size depicted in the patient’s preoperative photographs of her desired breast size.

Once the implant volume was identified, the implants were selected, soaked in triple antibiotic solution, and placed in the submuscular pocket. Final implant position was adjusted with the patient sitting upright to get optimal implant position and optimal symmetry between the two breasts.

### Mastopexy Surgical Technique

With the patient still in the sitting upright position, a Tailor-Tack mastopexy was done to approximate the Wise pattern mastopexy before making any incisions, using only staples. The staples were adjusted on both breasts to achieve optimal shape of both breasts and optimal symmetry between the two breasts. The nipple-areola complex was temporarily buried behind the staples. Mild intentional overcorrection creates convexity of the upper pole, and the lower pole of the breast was intentionally slightly tight and flattened. The “midequator” of the breast mound (ie, where the convexity of the upper pole changes to the convexity of the lower pole) was drawn. This was used to mark the height of the middle of the final nipple position.

Once optimal shape of both breasts was determined in the sitting position, the patient was laid supine. Markings were made where the staples were placed and the staples were removed. The remainder of the skin within the completed Wise pattern was resected as a full-thickness skin excision. Final closure was accomplished with buried dermal polydioxanone suture and running subcuticular Quills. The nipple-areola complex was brought out through a new circle, such that the center of the nipple would be at the “equator” of the breast mound (ie, at the junction of the superior pole and the inferior pole). Typically, B cup breasts have a vertical incision line, from the inframammary fold to the bottom of the areola, of approximately 5 cm; C cup breasts have a vertical incision line of approximately 7 cm; and D cup breasts have a vertical incision line of approximately 9cm. A 38-mm-diameter circle was excised in the breast mound centered on the breast equator and the nipple-areola complex was inset with buried interrupted polydiaxanone and running subcuticular Quill sutures. All wounds were postoperatively supported with steri-strips for 3 weeks.

## RESULTS

Fifty-six consecutively treated patients were identified as having concurrent mastopexy with augmentations during this 8-year period (Table 1 and **SDC1**; **see chart, Supplemental Digital Content 1**, which displays comorbidities, http://links.lww.com/PRSGO/B135). The average age of the studied patients was 41.2 years. The average follow-up time period was 2.1 years (±8.9 months). Fifty-four patients (96.4%) had implants placed through the periareolar approach. Two patients (3.6%) had implants placed through preexisting inframammary fold scars. All implants were placed submuscularly. Fifty-two patients (92.9%) received silicone implants and 4 patients (7.1%) received saline implants. Patient preference determined implant choice. All implants except 5 were textured. Implant sizes ranged from 120 to 800 cm^3^, and the average implant size was 277 cm^3^ (Table 2). Intraoperative Tailor-Tack mastopexies were performed on all patients. Twenty-two patients (39.3%) had additional nonbreast procedures performed along with the mastopexy and augmentation.

**Table 1.**
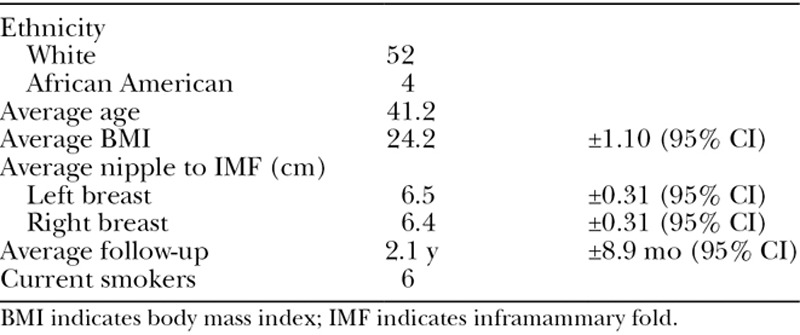
Demographic Data

**Table 2.**
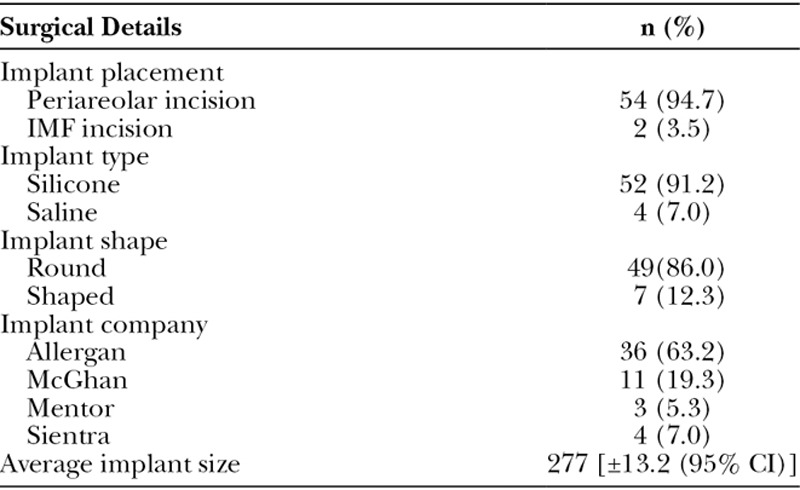
Surgical Details

A total of 10 patients had complications (17.9%), which were subdivided into tissue-related complications (16.1%) and implant-related complications (8.9%; Table 3). Only four patients (7.1%) who suffered complications had both tissue-related and implant-related complications. The majority of complications were tissue-related, which consisted of recurrent breast ptosis (3.6%), poor shape of the nipple areolar complex (7.1%), and hypertrophic scarring (8.9%). Smoking status, age, and BMI did not have a statistical significance regarding complication rates. Active smokers were not treated differently from nonsmokers because no undermining of the lateral breast flaps is required and the neurovascular supply of the nipple is the entire gland. Of note, 1 patient had both a hypertrophic scar and recurrent ptosis, and another patient had both a hypertrophic scar and a poor shape of the nipple areolar complex. There were neither early complications nor any cases of nipple loss, breast skin loss, or decreased nipple sensation.

**Table 3.**
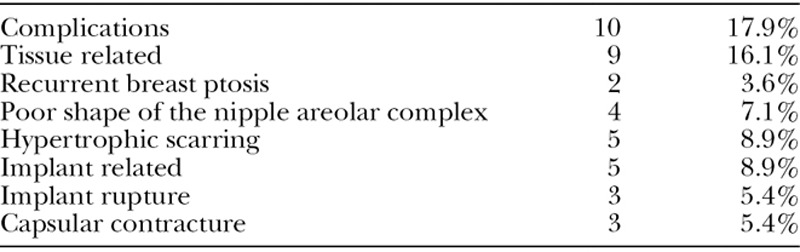
Complications

Implant-related complications (8.9%) consisted of implant rupture (5.4%) and capsular contracture (5.4%). One patient had both implant rupture and capsular contracture. There were no implant infections.

Fifty-three patients (94.6%) reported satisfaction with their surgical results. There was an overall 12.5% surgical revision rate, with 6 patients treated in the operating room and 1 patient in the office. One patient requested that her implants be permanently removed after quickly developing Baker III capsular contractures. Four patients (7.1%) required steroid injections with triamcinolone for hypertrophic scars.

## DISCUSSION

**Fig. 1.**
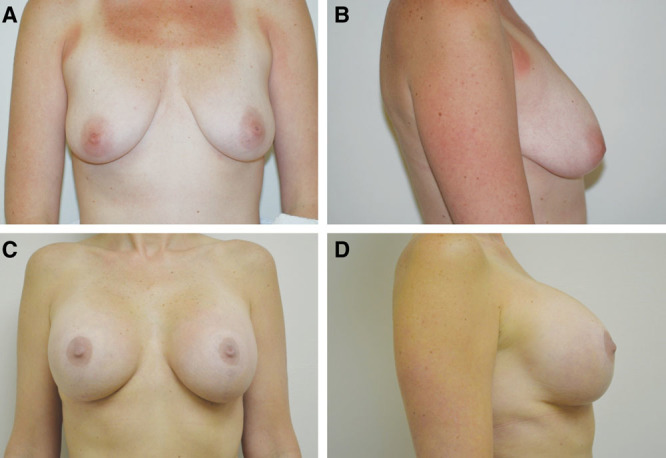
Case example of 37-year-old woman with grade II ptosis seeking increased volume and lift. A and B, Preoperative images. C and D, Postoperative result at 2 years and 10 months. The breast volume was increased with textured, 270 cm^3^ Allergan style 110, silicone implants placed submuscularly.

**Fig. 2.**
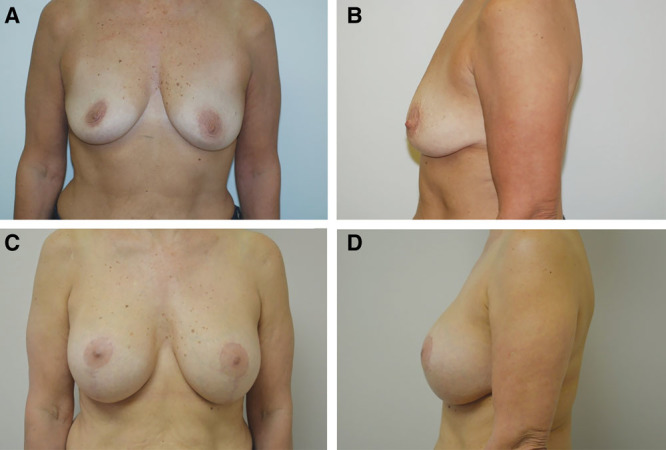
Case example of 59-year-old woman with grade II ptosis, seeking increased volume and lift. A and B, Preoperative images. C and D, Postoperative result at 2 years and 2 months. The breast volume was increased with textured, 354 cm^3^ Allergan style 115 silicone implants, placed submuscularly.

Despite decades of debate, many surgeons still question the safety of concurrently performing mastopexy and augmentation. The data from this study suggest that single-stage mastopexy with augmentation is an acceptably safe and reproducible surgical technique. The technique can be applied to women of varying ages and/or degrees of ptosis; ptosis is corrected; volume is expanded; and the nipple neurovascularity remains uncompromised (Figs. 1, 2).

Comparing this study’s complications to national averages, the complication rates with the single-stage procedure are equivalent to or below the reported complication rates of isolated mastopexy, isolated breast augmentation, and mastopexy with augmentation. Overall, the complication rate was 17.9%, whereas other large studies document overall mastopexy with augmentation complication rates of 13%–36%.^6–10,12^ Recently, several surveyed physicians reported a 28.1% revision rate with mastopexy with augmentation. Nationally, the incidence of secondary surgery after breast augmentation ranges from 0% to 36% at 10 years.^13^ Incidence of revision with isolated mastopexy has been reported to be 14.6%; the reported rate has been as high as 26% when a circumareolar incision is used.^14^ The revision rate for this study was 12.5%. The technique had no implant infections, whereas the national average of implant infections with breast augmentation alone and with concurrent mastopexy are 2%–2.5% and 1.3%, respectively.^13,15,16^ Reported capsular contracture rates after primary submuscular breast augmentation rates range from 7.6% to 20%.^7,10,12^ Capsular contracture rates observed with mastopexy with augmentation rates have been cited from 5.9% to 8%.^8,12^ Our capsular contracture rate was 5.4%.

The “Tailor-Tack” mastopexy and the mastopexy with augmentation are not novel concepts. This study’s contribution to this concept is to stress that one should establish the new breast volume initially and then proceed with the mastopexy, as it is impossible to preoperatively determine skin resection when breast volume will ultimately change. Intraoperatively derived skin markings should be made after the new breast volume has been established. In the authors’ experience, this intraoperative planning for skin resection routinely results in a lower rate of complications and yields aesthetically pleasing results for the patient and surgeon. The authors agree with colleagues that there should be an algorithmic approach specific to each individual when developing a preoperative plan for mastopexy augmentation.^17^ However, the authors disagree with some colleagues in that they do not believe that any preoperative measurement or calculation of skin resection can be adequately precise. Once the implant is placed, all preoperative measurements lose validity, as the implant changes the breast dimensions, projection, and soft tissue tension. It is only after the volume change from implant placement that the skin and soft tissues can be precisely tailored to the new breast volume to achieve optimal aesthetic outcomes while protecting skin flaps, tension, and the nipple. This is exactly what a professional tailor does during the final fitting of a custom dress or suit.

## CONCLUSIONS

The authors believe that mastopexy can safely be performed concurrently with breast augmentation. In this 8-year review, there were no catastrophic complications such as skin loss, nipple loss, implant extrusion, or infection. The complications identified, such as recurrent breast ptosis, implant rupture, and capsular contracture, were the usual complications known to occur with independently performing mastopexy or breast augmentation. These issues occurred at rates comparable to or less than national averages for those procedures when they are performed independently. The authors believe that the paramount principle for the success of this technique is that breast volume should be adjusted first, followed with an intraoperatively determined skin resection that precisely fits the new breast volume. By adjusting the breast skin envelope to the new breast volume, one can concurrently perform mastopexy and breast augmentation without compromising the vascularity of the nipple areolar complex or breast skin and will achieve the desired lift and optimal aesthetic outcomes.

## Supplementary Material
