# Supplementary figures and images for: Can It Be Safe and Aesthetic? An Eight-year Retrospective Review of Mastopexy with Concurrent Breast Augmentation
Source: Plast Reconstr Surg Glob Open. 2019 Jun 12;7(6):e2272. doi: 10.1097/GOX.0000000000002272 (PMC6635184; doi:10.1097/GOX.0000000000002272)

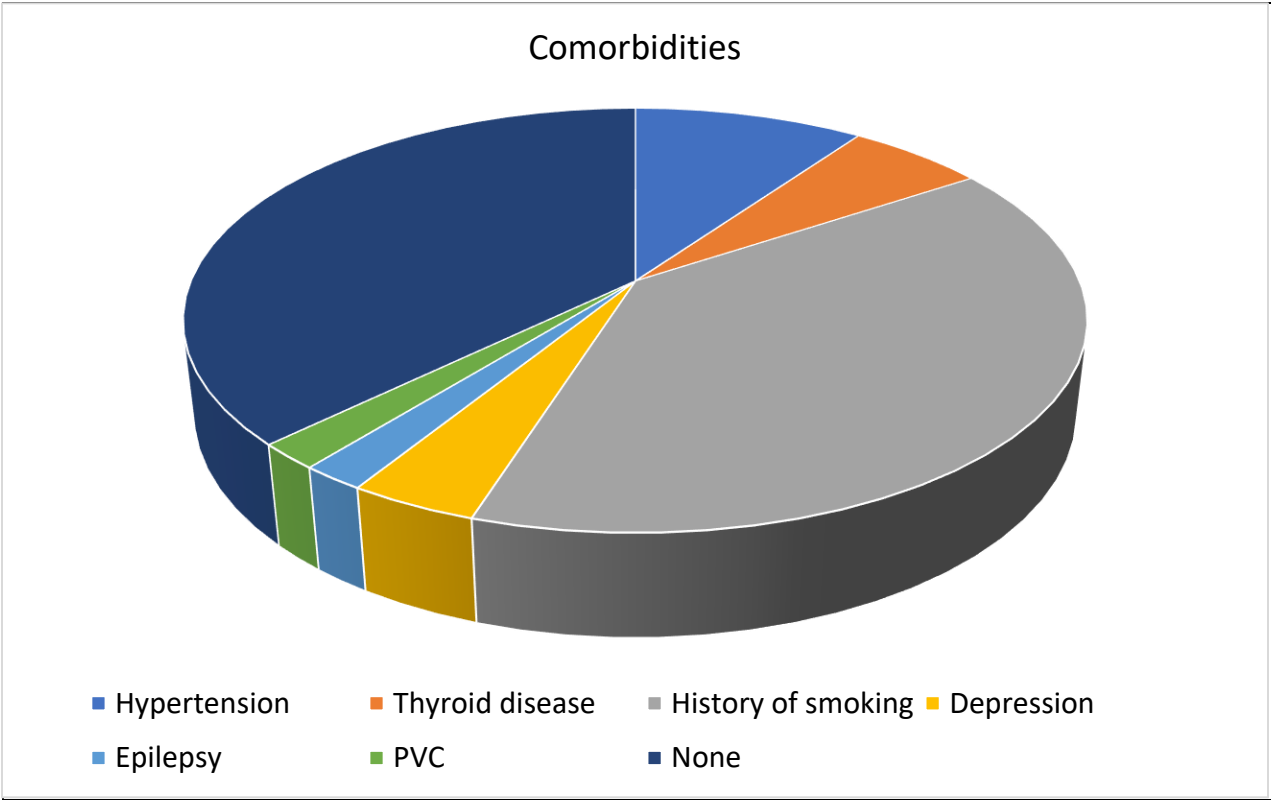

| Comorbidites       |    |        |
|--------------------|----|--------|
| Hypertension       | 5  | 8.93%  |
| Thyroid disease    | 3  | 5.36%  |
| History of smoking | 20 | 35.71% |
| Depression         | 2  | 3.57%  |
| Epilepsy           | 1  | 1.79%  |
| PVC                | 1  | 1.79%  |
| None               | 19 | 33.93% |

Supplement: Supplementary file 1 [file gox-7-e2272-s001.pdf]
